# Toll-like receptor 3 gene polymorphisms and severity of pandemic A/H1N1/2009 influenza in otherwise healthy children

**DOI:** 10.1186/1743-422X-9-270

**Published:** 2012-11-15

**Authors:** Susanna Esposito, Claudio Giuseppe Molteni, Silvia Giliani, Cinzia Mazza, Alessia Scala, Laura Tagliaferri, Claudio Pelucchi, Emilio Fossali, Alessandro Plebani, Nicola Principi

**Affiliations:** 1 Department of Pathophysiology and Transplantation, Università degli Studi di Milano, Fondazione IRCCS Ca’ Granda Ospedale Maggiore Policlinico, Via Commenda 9, Milano, 20122, Italy; 2 Nocivelli Institute for Molecular Medicine and Pediatric Clinic, University of Brescia, Brescia, Italy; 3 Department of Epidemiology, Istituto di Ricerche Farmacologiche Mario Negri, Milan, Italy; 4 Pediatric Emergency Unit, Fondazione IRCCS Ca’ Granda Ospedale Maggiore Policlinico, Milan, Italy

**Keywords:** Children, Innate immunity, Influenza, Pandemic A/H1N1/2009 influenza virus, TLR3, Toll-like receptors

## Abstract

**Background:**

Toll-like receptors (TLRs) form an essential part of the innate immune system, which plays a fundamental role in rapidly and effectively controlling infections and initiating adaptive immunity. There are no published data concerning the importance of polymorphisms of TLRs in conditioning susceptibility to influenza or the severity of the disease. The aim of this study was to evaluate whether selected polymorphisms of TLR2, TLR3 and TLR4 influence the incidence and clinical picture of pandemic A/H1N1/2009 influenza.

**Results:**

The study involved 272 healthy children attending our Emergency Room for influenza-like illness (ILI), including 51 (18.8%) with pandemic A/H1N1/2009 influenza as revealed by real-time polymerase chain reaction, and 164 healthy controls examined after minor surgery. Genomic DNA was extracted from whole blood samples and five single-nucleotide polymorphisms (SNPs) were studied: TLR2 rs5743708, TLR3 rs5743313, TLR3 rs5743315, TLR4 rs4986790 and TLR4 rs4986791. The TLR3 rs5743313/CT polymorphism was found in all of the children with pneumonia and influenza infection, but in a significantly smaller number of those with A/H1N1/2009 influenza without pneumonia (<0.0001). TLR2, TLR3 rs5743315/AC and TLR4 polymorphisms were equally distributed in all of the groups regardless of the presence of the pandemic A/H1N1/2009 virus and clinical diagnosis. Viral load was comparable in all of the study groups.

**Conclusions:**

There is a close relationship between the presence of TLR3 rs5743313/CT and an increased risk of pneumonia in children infected by the pandemic A/H1N1/2009 influenza virus.

## Background

Toll-like receptors (TLRs) are a family of transmembrane proteins expressed on many cells, including dendritic and natural killer cells, macrophages and the cells of the respiratory epithelium. They form an essential part of the innate immune system, which plays a fundamental role in rapidly and effectively controlling infections and initiating adaptive immunity [1-8]. The central role of TLR activation in both innate and adaptive immune responses is demonstrated by the fact that TLR polymorphisms influence susceptibility to a number of viral pathogens as well as the severity of the diseases they cause [9-14].

It has been shown that TLR3 and TLR7 make the main contribution to innate defences against influenza viruses [15,16], but there are few published data concerning the importance of polymorphisms of these and other TLRs in conditioning susceptibility to influenza or the severity of the disease [17,18]. However, such information is important because it could allow the identification of subjects for whom specific preventive and therapeutic interventions should be planned in order to reduce the clinical and socio-economic impact of influenza infection. Moreover, a knowledge of how innate immunity confronts influenza viruses could contribute to the development of significantly more effective vaccines and drugs to prevent and treat the disease.

An A/H1N1 quadruple reassortant influenza virus (A/H1N1/2009) of swine origin has recently arisen from an A/H1N1 influenza virus subtype that was already endemic in humans [19]. The aim of this study was to evaluate whether selected polymorphisms of TLR2, TLR3 and TLR4 (chosen from among those previously associated with modified signalling function and a greater susceptibility to presumed or demonstrated infection) influence the incidence and clinical picture of the disease caused by this virus.

## Results

The study involved 436 prospectively and consecutively enrolled children: 272 with influenza-like illness (ILI), including 51 (18.8%) who were positive for pandemic A/H1N1/2009 virus, and 164 healthy controls. None of the blood cultures of the children with ILI was positive for bacteria. Table 1 shows the demographic and clinical characteristics of the children in the three groups. There were no between-group differences in terms of gender, race, age distribution, the number of siblings, exposure to passive smoking, day care or school attendance, or previous pneumococcal and/or pandemic influenza vaccination. Moreover, the distribution of the clinical diagnoses and the rates of hospitalisation were similar in both etiological groups of patients with ILI. All of the patients with ILI symptoms and a diagnosis of pneumonia were hospitalised; all of the others were discharged.

**Table 1:** Demographic and clinical characteristics of the study population

**Characteristics**	**A/H1N1/2009-positive children admitted because of ILI(n=51)**	**A/H1N1/2009-negative children admitted because of ILI(n=221)**	**Healthy controls(n=164)**
Males, No. (%)	27 (52.9)	109 (49.3)	85 (51.8)
Caucasians, No. (%)	51 (100.0)	221 (100.0)	164 (100.0)
Mean age ± SD, yrs	**3**.**16** ± **3**.**46**	3.43 ± 3.79	3.39 ± 3.88
Age groups, No. (%)			
<2 years	**5** (**9**.**8**)	28 (12.7)	15 (9.1)
2-5 years	**44** (**86**.**3**)	182 (82.4)	140 (85.4)
>5 years	**2** (**3**.**9**)	11 (5.0)	9 (5.5)
Median number of siblings (range)	1 (0–4)	1 (0–3)	1 (0–4)
Exposure to passive smoking, No. (%)	15 (29.4)	69 (31.2)	43 (26.2)
Day care or school attendance, No. (%)	49 (96.1)	210 (95.0)	158 (96.3)
Previous pneumococcal vaccination, No. (%)	46 (90.2)	199 (90.0)	143 (87.2)
Previous pandemic A/H1N1/2009 influenza vaccination, No. (%)	0 (0.0)	3 (1.4)	2 (1.2)
Diagnosis, No. (%)			
Common cold	15 (29.4)	78 (35.2)	NA
Pharyngitis	14 (27.5)	75 (33.9)	NA
Acute otitis media	4 (7.8)	21 (9.5)	NA
Pneumonia	18 (35.4)	47 (21.2)	NA
Minor surgical problem	NA	NA	164 (100.0)
Hospitalised at enrolment, No. (%)	18 (35.4)	47 (21.2)	NA

ILI: influenza-like illness; NA: not applicable; SD: standard deviation. Percentages in parentheses. No significant between-group difference.

Table 2 shows the genotype frequency of the selected TLR polymorphisms in the study population as a whole. There was no significant difference in their frequency between the children who were positive or negative for pandemic A/H1N1/2009 influenza, or between either of the etiological groups of children with ILI and the healthy controls.

**Table 2:** **Genotype frequency of toll**-**like receptor** (**TLR**) **polymorphisms in the study population**

**Polymorphism genotypes**	**A/H1N1/2009-positive children admitted because of ILI(n=51)**	**A/H1N1/2009**-**negative children admitted because of ILI(n=221)**	**Healthy controls(n=164)**
TLR2 rs5743708			
GG	49 (96.1)	218 (98.6)	161 (98.2)
GA	2 (3.9)	3 (1.4)	3 (1.8)
*p*-value, HWE	0.89	0.92	0.91
TLR3 rs5743313			
CC	26 (51.0)	124 (56.1)	92 (56.1)
TT	4 (7.8)	12 (5.4)	9 (5.5)
CT	21 (41.2)	85 (38.5)	63 (38.4)
*p*-value, HWE	0.93	0.60	0.67
TLR3 rs5743315			
CC	46 (90.2)	208 (94.1)	149 (90.9)
AC	5 (9.8)	13 (5.9)	15 (9.1)
*p*-value, HWE	0.71	0.65	0.54
TLR4 rs4986790			
AA	46 (90.2)	202 (91.4)	148 (90.2)
AG	5 (9.8)	19 (8.6)	16 (9.8)
*p*-value, HWE	0.71	0.50	0.51
TLR4 rs4986791			
CC	46 (90.2)	203 (91.9)	148 (90.2)
TT	0 (0.0)	1 (0.5)	0 (0.0)
CT	5 (9.8)	17 (7.6)	16 (9.8)
*p*-value, HWE	0.71	0.33	0.51

HWE: Hardy-Weinberg equilibrium; ILI: influenza-like illness; TLR: toll-like receptor. Percentages in parentheses. No significant between-group difference.

Table 3 shows the genotype frequency of the TLR polymorphisms in the children who were positive for A/H1N1/2009 influenza virus, by disease severity. The TLR2, TLR3 rs5743315/AC and TLR4 polymorphisms were equally distributed in the two groups regardless of the clinical diagnosis, whereas the TLR3 rs5743313/CT polymorphism was found in all of the children with pneumonia and influenza infection, but in a significantly smaller number of those A/H1N1/2009 influenza without pneumonia without pneumonia (<0.0001). Viral load was comparable in all of the study groups.

**Table 3:** **Genotype frequency of toll**-**like receptor** (**TLRs**) **polymorphisms in children with pandemic A**/**H1N1 influenza infection by disease severity**

**Polymorphism genotypes**	**A/H1N1/2009-positive children hospitalised because of pneumonia(n=18)**		**A/H1N1/2009-positive children discharged with a diagnosis other than pneumonia(n=33)**	
	**Frequency**	**Viral load (mean log**_**10**_**cp/mL ± SD)**	**Frequency**	**Viral load (mean log**_**10**_**cp/mL ± SD)**
TLR2 rs5743708				
GG	17 (94.4)	7.43 ± 1.30	32 (97.0)	7.06 ± 1.57
GA	1 (5.6)	3.88	1 (3.0)	8.29
*p*-value, HWE	0.90		0.93	
TLR3 rs5743313				
CC	0 (0.0)*	NA	26 (78.8)	6.94 ± 1.45
TT	0 (0.0)	NA	4 (12.1)	7.24 ± 2.42
CT	18 (100.0)*	7.23 ± 1.51	3 (9.1)	8.07 ± 1.33
*p*-value, HWE	<0.001		<0.001	
TLR3 rs5743315				
CC	15 (83.3)	7.26 ± 1.66	31 (93.9)	7.12 ± 1.59
AC	3 (16.7)	7.10 ± 0.36	2 (6.1)	6.69 ± 1.25
*p*-value, HWE	0.70		0.86	
TLR4 rs4986790				
AA	16 (88.9)	7.12 ± 1.53	30 (90.9)	7.00 ± 1.56
AG	2 (11.1)	8.14 ± 1.41	3 (9.1)	8.01 ± 1.57
*p*-value, HWE	0.80		0.78	
TLR4 rs4986791				
CC	17 (94.4)	7.24 ± 1.56	29 (87.9)	7.00 ± 1.56
TT	0 (0.0)	NA	0 (0.0)	NA
CT	1 (5.6)	7.14	4 (12.1)	8.01 ± 1.57
*p*-value, HWE	0.90		0.71	

HWE: Hardy-Weinberg equilibrium; NA: not available; TLR: toll-like receptor. Percentages in parentheses. *p<0.0001 *vs* A/H1N1/2009-positive children discharged with a diagnosis other than pneumonia; no other significant between-group difference.

## Discussion

In this study, we found that the prevalence of selected TLR2, TLR3 and TLR4 polymorphisms potentially associated with an increased risk of infection was similar in healthy children and in patients with ILI, regardless of whether they were positive or negative for pandemic A/H1N1/2009 influenza virus infection. This means that the occurrence of influenza does not seem to depend on these genetic variants. The same conclusion could be drawn regarding the risk of superimposed bacterial infections, because the cultured blood of all of the children with ILI was negative for bacteria at the time of diagnosis, regardless of the etiology of the disease. However, this merits further study because blood cultures are not very sensitive for the detection of bacteremia, and only molecular methods can exclude co-infection with viral and bacterial respiratory pathogens [20].

We found a close relationship between the presence of the TLR3 rs5743313/CT polymorphism and an increased risk of pneumonia in children infected by the pandemic A/H1N1/2009 influenza virus. As the children with pandemic A/H1N1/2009 influenza who were hospitalised for pneumonia were similar to those with pandemic A/H1N1/2009 influenza who were discharged from the Emergency Room because their disease was milder, this finding suggests that the TLR3 rs5743315/AC polymorphism *per se* may play a role in conditioning the risk of pneumonia in children infected by pandemic A/H1N1 influenza virus. The fact that viral load was not higher in the children with this genetic variation does not modify this conclusion because viral load is not a reliable expression of the severity of viral illness. Viral load declines over time and, because the respiratory secretions used to evaluate it were not always collected at the beginning of the disease, it may not correlate with the clinical picture [21,22].

Although TLR3 is considered an essential part of the innate immune system, it is abundantly expressed in dendritic cells, recognises viral pathogens, and induces interferon beta (IFN-b) production [7]. It is likely that the antiviral effects of TLR3 signalling on influenza virus infection are mediated by the stimulation of a variety of cells (such as hematopoietic stem cells, monocyte-derived dendritic cells, endothelial cells and NK cells) to produce type I IFN, which subsequently inhibits viral replication. However, whether it protects or not against influenza viruses has not been precisely defined. In experimental animals, the administration of TLR3 agonists is effective in reducing the damage caused by influenza [23,24] because they inhibit the replication of mouse-adapted laboratory influenza A viruses and clinical isolates from humans (including considerably virulent strains with pandemic potential). Moreover, the induction of local pulmonary inflammation such as that caused by TLR3 activation has been found to protect against influenza infection in mice [25]. It has also been found that TLR3 activation in the context of an influenza virus infection leads to excess cytokine production and inflammatory lung alterations that cause severe tissue damage and reduce survival in TLR3-deficient mice, [26]. The reasons given to explain these conflicting findings include the type of virus used to infect the animals, viral load, the infected cell type, and the stage of infection [27].

However, our data seem to suggest that, although TLR3 does not affect the occurrence of pandemic A/H1N1/2009 influenza, it might favourably condition the clinical course of the disease because the incidence of influenza-related pneumonia was significantly higher in the children with TLR3 polymorphisms than in those without. As the polymorphism associated with a more severe course of pandemic A/H1N1/2009 influenza (TLR3 rs5743313/CT) is located near exon 4 (i.e. the gene region encoding trans-membrane signal induction), it may play a significant role in reducing the efficiency of the mechanisms that recognise influenza virus, thus leading to cytokine production and a reduction in immune defences that favours more severe influenza. This hypothesis seems to be supported by the findings that exon 4 in the gene of TLR3 is very rich in tyrosine, a target of the tyrosine kinase c-SRC, and that an association between TLR3 and c-SRC tyrosine kinase on endosomes is essential to initiate antiviral signalling [28].

Associations between TLR3 polymorphisms and human diseases have been frequently demonstrated. Variations in the TLR3 gene may be found in patients with type 1 diabetes, Steven-Johnson syndrome, and toxic epidermal necrolysis [27,29]. In relation to viral infections, Zhang *et al*. identified a dominant-negative TLR3 allele in patients with herpes simplex virus 1 (HSV-1) encephalitis [9], and Bell *et al*. found a heterozygous mutation (Phe554Ser) associated with impaired TLR3-dependent interferon induction in response to HSV-1 [10]. TLR3 polymorphisms have also been found in Japanese subjects with sub-acute sclerosing panencephalitis due to measles virus [11]. In addition, a missense mutation of TLR3 has been detected in a patient with influenza-associated encephalopathy [17]. Finally, a recent study regarding TLR3 polymorphisms and their association with hepatitis B virus (HBV) infection [18] found that whereas genetic variance (rs1879026/GT) was more common in HBV carriers than in uninfected controls haplotype analysis revealed that specific haplotypes GCGA (rs1879026, rs5743313, rs5743314, and rs5743315) were significantly associated with HBV infection.

This study has the limitation that only a part of the TLR3 gene was studied, and so it is not possible to establish whether the presence of other genetic variants involving TLR3 can explain our findings. However, although based on a partial evaluation of the genetic characteristics of TLR3, our data add further information concerning the relationships between TLR3 and the development of diseases, and offer further support for identifying subjects at increased risk of influenza-related complications, and developing new preventive and therapeutic measures against influenza. The recent finding that the administration of a TLR3-mucosal adjuvant together with an intranasal influenza vaccine can significantly improve immune response by inducing cross-protective mucosal immunity against heterologous influenza virus infection is the best demonstration of how a better knowledge of the role of innate immunity against influenza viruses can increase our chances of reducing the clinical and socio-economic impact of this common disease [30-32].

## Conclusions

Our findings show that there is a close relationship between the presence of TLR3 rs5743313/CT and an increased risk of pneumonia in children infected by the pandemic A/H1N1/2009 influenza virus, whereas the prevalence of other selected TLR2, TLR3 and TLR4 polymorphisms does not seem to be associated with an increased risk of infection.

## Methods

### Ethics statement

This study was approved by the Institutional Review Board of Fondazione IRCCS Ca’ Granda Ospedale Maggiore Policlinico, Milan, Italy, and was carried out in the Department of Maternal and Pediatric Sciences of the University of Milan between 15 October and 30 November 2010. The written informed consent of both parents or a legal guardian was required, and the older children were asked to give their assent.

### Study subjects

This prospective study involved consecutively enrolled, otherwise healthy subjects aged less than 15 years who visited the Emergency Room of the Department because of an influenza-like illness (ILI) as defined by the Italian Ministry of Health (http// http://www.ministerosalute.it): an acute respiratory disease of sudden onset, with fever (an axillary temperature of >38°C), accompanied by at least one of the general symptoms of headache, generalised malaise, a feverish sensation (sweating and chills) or asthenia, and one of the respiratory symptoms of cough, pharyngodynia or nasal congestion. The exclusion criteria were chronic diseases increasing the risk of complications of viral respiratory infections, including premature birth; chronic disorders of the pulmonary or cardiovascular systems, including asthma; chronic metabolic diseases, including diabetes mellitus; neoplasia; kidney or liver dysfunction; hemoglobinopathies; immunosuppression; diseases requiring long-term aspirin therapy; and genetic or neurological disorders.

After a complete physical examination, the children were divided into disease groups on the basis of their signs and/or symptoms using well-established criteria [33]. All of the children underwent a chest X-ray, and the radiological findings were evaluated by an independent radiologist blinded to the other study variables. The children with radiographically confirmed pneumonia as defined by the WHO [34] were hospitalised and considered as having severe disease. Those with a diagnosis other than pneumonia were sent home and considered as having mild disease.

Upon enrolment, a nasopharyngeal sample was collected for the diagnosis of pandemic A/H1N1/2009 influenza infection, and a blood sample was drawn for bacterial culture and genetic analysis.

As a control group, a similar number of healthy children selected from those consecutively admitted to the Department for a control examination following minor surgery during the same study period were genetically analysed.

### Laboratory assays

#### Identification of A/H1N1 influenza virus

A nasopharyngeal sample was collected from all of the children using a flexible pernasal flocked swab, and stored in a mini-tube containing 1 mL of universal transport medium (UTM-RT Kit Cat. No. 360c, Copan Italia, Brescia, Italy). The nasopharyngeal samples were collected from each child by trained pediatricians. The distance between the patient’s nares and ear lobe was measured to estimate the length of insertion, after which the swabs were gently inserted towards the pharynx until resistance was felt, and then rotated three times to obtain epithelial cells. They were then withdrawn and put into a tube containing the specific transport medium. All of the specimens were kept cool and delivered to the laboratory within three hours of collection. Viral RNA was extracted by means of a Nuclisens EasyMAG automated extraction system (bioMeriéux, Bagno a Ripoli, Florence, Italy) using a generic protocol, a 190 μL sample input, and 10 μL of cultured phocine distemper virus (PDV) (the extraction/PCR inhibition control was kindly provided by Prof. H.G.M. Niesters, UMCG, Groningen, The Netherland) [31,32]. A real-time polymerase chain reaction (real-time PCR) to identify A and B influenza viruses was performed using validated methods that have been previously described [35]. The primer/probe sets were: influenza A forward AAGACCAATCCTGTCACCTCTGA, reverse CAAAGCGTCTACGCTGCAGTCC, probe FAM-TTTGTGTTCACGCTCACCGTGCC-BHQ1; influenza B forward GAGACACAATTGCCTACCTGCTT, reverse TTCTTTCCCACCGAACCAAC, probe TET-AGAAGATGGAGA AGGCAAAGCAGAACTAGC-Eclipse; PDV forward CGGGTGCCTTTTACAAGAAC, reverse TTCTTTCCTCAACCTCGTCC, probe VIC-ATGCAAGGGCCAATTCTTCCAAGTT-BHQ1. In the case of influenza A-positive samples, the WHO/CDC protocol was used to characterise pandemic A/H1N1/2009 [35] with the following primers/probes: SWInfA forward GCACGGTCAGCACTTATYCTRAG; SWInfA reverse GTGRGCTGGGTTTTCATTTGGTC; SWInfA probe FAM-CYACTGCAAGCCCA’T’ACACACAAGCAGGCA; SWH1 forward GTGCTATAAACACCAGCCTYCCA; SWH1 reverse CGGGATATTCCTTAATCCTGTRGC; SWH1 probe CAGAATATACA’T’CCRGTCACAATTGGARAA (‘T’=3’ modified to prevent extension by TAQ polymerase and linked to BHQ1).

A recombinant plasmid construct carrying the corresponding HA target sequence (kindly provided by Fausto Baldanti of the Molecular Virology Unit, Fondazione IRCCS Policlinico San Matteo, Pavia, Italy) was used to quantify viral load in the influenza A/H1N1/2009-positive samples. In detail, ten-fold plasmid serial dilutions ranging from 5 to 5 x 10^7^ input copies were included in each assay run in order to allow the construction of run-specific calibration curves that would accurately quantify viral load in each of the archived samples. The cycle threshold (Ct) values of each dilution were measured in duplicate and plotted against the logarithm of their initial quantities, and the copy numbers in each clinical sample were derived from the regression line. The quantitative results were expressed as RNA copy number/mL of nasopharyngeal swab following data multiplication by 50 (dilution factor). In order to evaluate reproducibility, intra- and inter-assay standard deviations (SDs) and coefficients of variation were calculated for each standard concentration within and between the individual PCR runs.

#### Blood cultures

The blood was cultured using a BacT/ALERT (bioMérieux, Florence, Italy) or BacTec 9240 (Becton Dickinson, Buccinasco, Italy).

#### Genotype analyses

The erythrocyte/granulocyte fraction was isolated from whole blood samples using Histopaque-1077 (Sigma-Aldrich, Milan, Italy), and genomic DNA was extracted from this fraction using a Nuclisens EasyMAG automated extraction system (Biomeriéux), the whole blood (Specific B) protocol, and a 100 μL sample input volume.

The studied single-nucleotide polymorphisms (SNPs) were selected on the basis of their previously reported associations with different diseases, preferably of infectious origin. In the case of the TLR2 and TLR4 genes, these were non-synonymous SNPs because of their association with tuberculosis (TLR2 Arg753Gln [rs5743708]) [36-38] and respiratory syncytial virus infection (TLR4 Asp299Gly [rs4986790] and TLR4 Thr399Ile [rs4986791]) [13,14]. Two SNPs in the TLR3 gene (intron 3) were selected because of their proximity to exon 4, a tyrosine-rich region that is a target site of the tyrosine kinase c-SRC whose association with TLR3 in dsRNA-containing endosomes is necessary to initiate antiviral signalling [26]. The SNPs were genotyped on genomic DNA using pre-designed Taqman® SNP Genotyping assays, with the exception of the TLR3 2593 C/T polymorphism, for which a Custom Taqman® SNP Genotyping assay (Applied Biosystems, Monza, Italy) was designed using the following primers/probes: 5’-CATTGGTGTCATCCTCCTGAGA-3’ (forward), 5’-GCAGGGCGGCAGAGT-3’ (reverse), VIC-TCTCCCGACCTCTCC-MGBNFQ (probe 1), FAM-TCTCCCAACCTCTCC-MGBNFQ (probe 2) (Applied Biosystems) [36]. The reactions and allelic discrimination analyses were performed using an ABI PRISM 7000 sequence detection system (Applied Biosystems). Reference samples for each genotype were included in all of the reaction plates, and the genotypes were confirmed by sequencing.

### Statistical analyses

The data were analysed using SAS for Windows v. 9.1 (SAS Institute, Cary, NC, USA). The continuous variables are given as mean values ± standard deviation, and were analysed using a non-parametric test (i.e. the two-sided Wilcoxon’s rank-sum test) as they were not normally distributed (on the basis of the Shapiro-Wilk statistic); the categorical variables are given as numbers and percentages, and were analysed using contingency tables and the chi-squared or Fisher’s test, as appropriate. Allele and genotype frequencies were calculated by direct counting. In order to investigate Hardy-Weinberg equilibrium (HWE), we computed the expected numbers of different genotypes under HWE and compared them with the observed numbers, and then assessed potential deviations using chi-squared tests.

## Abbreviations

CT: Cycle threshold; HWE: Hardy-Weinberg equilibrium; ILI: Influenza-like illness; PDV: Phocine distemper virus; SD: Standard deviation; DNPs: Single-nucleotide polymorphisms; TLR: Toll-like receptor.

## Competing interests

The authors have no potential conflict of interest to declare.

## Authors’ contributions

SE and NP designed the study and co-wrote the manuscript. CGM, SG, CM and AS carried out the laboratory assays. LT visited the hospitalised patients, collected the swabs, and entered the data in the database. CP statistically analysed the data. EF examined the patients in the Emergency Room. AP supervised the laboratory assays. All of the authors read and approved the final manuscript.
